# Study of liquid-phase molecular packing interactions and morphology of fatty acid methyl esters (biodiesel)

**DOI:** 10.1186/s13068-014-0194-7

**Published:** 2015-02-04

**Authors:** Paula Berman, Nitzan Meiri, Luiz Alberto Colnago, Tiago Bueno Moraes, Charles Linder, Ofer Levi, Yisrael Parmet, Michael Saunders, Zeev Wiesman

**Affiliations:** The Phyto-Lipid Biotechnology Laboratory, Departments of Biotechnology, Energy and Environmental Engineering, Ben-Gurion University of the Negev, P.O. Box 653, Beer-Sheva, 84105 Israel; Embrapa Instrumentação, Rua 15 de Novembro 1452, São Carlos, SP 13560-970 Brazil; Instituto de Física de São Carlos, Universidade de São Paulo, Av. Trabalhador Sao-Carlense 400, São Carlos, SP 13566-590 Brazil; Zuckerberg Center for Water Sciences and Technology and Department of Biotechnology, Ben-Gurion University of the Negev, P.O. Box 653, Beer-Sheva, 84105 Israel; Department of Industrial Engineering and Management, Ben-Gurion University of the Negev, P.O. Box 653, Beer-Sheva, 84105 Israel; Department of Management Science and Engineering, Stanford University, Stanford, CA USA

**Keywords:** ^1^H low field nuclear magnetic resonance relaxometry, Biodiesel physical properties, Methyl oleate, Molecular packing, Oleic acid, Segmental motion

## Abstract

**Background:**

^1^H low field nuclear magnetic resonance (LF-NMR) relaxometry has been suggested as a tool to distinguish between different molecular ensembles in complex systems with differential segmental or whole molecular motion and/or different morphologies. In biodiesel applications the molecular structure versus liquid-phase packing morphologies of fatty acid methyl esters (FAMEs) influences physico-chemical characteristics of the fuel, including flow properties, operability during cold weather, blending, and more. Still, their liquid morphological structures have scarcely been studied. It was therefore the objective of this work to explore the potential of this technology for characterizing the molecular organization of FAMEs in the liquid phase. This was accomplished by using a combination of supporting advanced technologies.

**Results:**

We show that pure oleic acid (OA) and methyl oleate (MO) standards exhibited both similarities and differences in the ^1^H LF-NMR relaxation times (T_2_s) and peak areas, for a range of temperatures. Based on X-ray measurements, both molecules were found to possess a liquid crystal-like order, although a larger fluidity was found for MO, because as the temperature is increased, MO molecules separate both longitudinally and transversely from one another. In addition, both molecules exhibited a preferred direction of diffusion based on the apparent hydrodynamic radius. The close molecular packing arrangement and interactions were found to affect the translational and segmental motions of the molecules, as a result of dimerization of the head group in OA as opposed to weaker polar interactions in MO.

**Conclusions:**

A comprehensive model for the liquid crystal-like arrangement of FAMEs in the liquid phase is suggested. The differences in translational and segmental motions of the molecules were rationalized by the differences in the ^1^H LF-NMR T_2_ distributions of OA and MO, which was further supported by ^13^C high field (HF)-NMR spectra and ^1^H HF-NMR relaxation. The proposed assignment allows for material characterization based on parameters that contribute to properties in applications such as biodiesel fuels.

## Background

Biodiesel production has increased dramatically over the last decade, raising the need for new rapid and non-destructive analytical tools and technologies. Several ^1^H low field nuclear magnetic resonance (LF-NMR) applications have been suggested by the authors for the field of biodiesel, including characterization of new alternative biodiesel resources by direct analysis of raw material sources, monitoring of the biodiesel transesterification reaction, and quality evaluation of the final product [1-6]. ^1^H LF-NMR relaxometry involves the measurement of relaxation constants, T_1_ and T_2,_ as a consequence of interactions between nuclear spins and their surroundings and among nuclear spins [6]. It was also suggested that the application of a novel numerical optimization method for analyzing ^1^H LF-NMR data [6,7] provides better resolved relaxation time distributions and more accurate solutions compared with those shown by existing numerical tools. For example, using this optimization method, the relaxation time distributions of rapeseed oil and biodiesel samples revealed four and three peaks, respectively, compared to a broad bimodal distribution and a single wide peak distribution for the same samples analyzed using WinDXP software [8].

^1^H LF-NMR spin-spin (T_2_) relaxometry has been suggested as a tool to distinguish between molecular populations in complex systems with differential mobilities and/or microscopic compartmentalization [9-13]. Still, the peaks in the analyzed T_2_ distributions of lipids have not been hitherto assigned to the appropriate molecular population arrangements with certainty. Marigheto *et al*. [14] speculated that the analyzed bimodal T_2_ distribution of an avocado oil sample arises from molecules of differing mobility, such as the oleic and palmitic constituents, or from nonequivalent proton pools of different mobility, such as those on methyl and olefinic groups. Adam-Berret *et al*. [15] found a similar two-peak distribution for tricaprin in the melt state, and suggested that this may be due to inhomogeneous relaxation rates for the protons along the side chains, or inhomogeneous organization of the triglycerides (TGs) in the liquid with intermolecular interactions. Of course, these hypotheses are interrelated, since different mobilities along the side chains of the TGs in the liquid phase are characteristic of different organizations and vice versa. Callaghan [16] studied the molecular motion of tristearin in the melt and found different T_2_s along the chains, which in turn were used to explain the tuning fork molecular configuration.

The liquid morphological structures of lipids, as opposed to crystal structures, have not been significantly studied because their experimental determinations are very difficult and require a combination of different investigation methods. Three models have been previously suggested for the arrangement of TGs in the melt, the smectic, nematic, and discotic liquid crystal models, as reviewed by Iwahashi and Kasahara [17]. However, a conclusive structure of liquid TGs still requires further research. Fatty acids (FAs) are significant building blocks of most lipids, including TGs. Short-range order was also found to exist between the aliphatic chains in the liquid state of FAs. This was attributed by Small [18] to a relatively small volume increase occurring during the melting of the crystalline chains to liquids, and was strengthened by the fact that X-ray scattering showed that domains of layered structures, with one dimension roughly equivalent to the lengths of the molecules, are present in the liquid.

The group of Iwahashi has thoroughly studied the self-organization of FAs in the neat liquid state [17,19-25]. They concluded, using near-infrared spectroscopy and vapor pressure osmosis on various FAs, that these exist mostly as dimers, even at high temperature, where the dimers are the units in their intra- or intermolecular movements. The dimers were found to aggregate to form clusters possessing the structure of a quasi-smectic liquid crystal, where the long-chained FA dimers arrange longitudinally and alternately to make an interdigitated structure in the clusters, with the tail of two dimers near the interacting head groups of the adjacent one. This has been determined from measurements of viscosity, density, high field (HF)-NMR, and X-ray diffraction [26].

The physical properties of FAs and their derivatives are largely determined by the length of the hydrocarbon chain and the degree of unsaturation, which affect the different degrees of molecular packing. In fully saturated compounds, free rotation around each carbon-carbon bond gives the hydrocarbon chain greater flexibility; the most stable conformation is the fully extended form, in which the steric hindrance of neighboring atoms is minimized. These molecules can pack together tightly in nearly crystalline arrays, with atoms all along their lengths in van der Waals contact with the atoms of neighboring molecules. In unsaturated FAs, a cis double bond forces a kink in the hydrocarbon chain. FAs with several such kinks cannot pack together as tightly as one-kink or fully saturated FAs, and their intermolecular interactions are therefore weaker. FAs of the same chain length have lower melting points as the degree of unsaturation is increased, as it takes less thermal energy to disorder them [27].

Surprisingly, the liquid structure of fatty acid methyl esters (FAMEs), which are derivatives of FAs, has attracted very little attention in the literature. FAMEs, the basic molecules that constitute biodiesel, can be achieved by transesterification of TGs using methanol in the presence of a catalyst. Their molecular organization in the melt is of high importance to the field of biodiesel, as it determines physico-chemical properties of the fuel, including flow properties, operability during cold weather, blending, and more.

Knothe [28] analyzed the cetane number, heat of combustion, cold flow, oxidative stability, viscosity, and lubricity of common FAMEs, and showed that oleic acid methyl ester (methyl oleate) is the best FAME for high quality biodiesel. Furthermore, there are several genetically modified oil seeds available in the market (soybean, sunflower, peanut) with high methyl oleate content (80%).

It was therefore the objective of this work to explore the potential of ^1^H LF-NMR spin-spin relaxometry technology to study the molecular details and aggregation of FAs and their FAME lipid derivatives in the liquid phase, using oleic acid and oleic acid methyl ester as a model biodiesel. This was accomplished by using a combination of supporting advanced technologies, including ^1^H LF-NMR diffusiometry, X-ray diffraction, and ^13^C and ^1^H HF-NMR. As will be shown, this new application of ^1^H LF-NMR is of high importance to the field of biodiesel characterization and also to other research and applied disciplines.

## Results

### ^1^H LF-NMR T_2_ distributions

**Figure 1:**
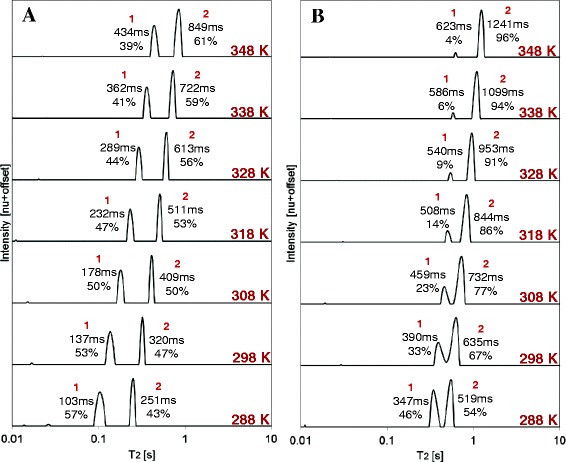
**Combined** ^**1**^ **H low field (LF)-NMR T**_**2**_ **distributions of (A) OA and (B) MO at different temperatures.** The relative contributions of each peak, in relation to other peaks and intrinsic T_2_ values, are shown on each plot. For the same temperature, the peaks in MO have larger T_2_ values compared to OA. As temperature increases, a shift in the T_2_ of the peaks is observed towards higher values, and the relative concentration of the peaks changes, especially for MO.

The combined ^1^H LF-NMR T_2_ distributions of oleic acid (OA) and methyl oleate (MO) at different temperatures are presented in Figure 1A and B, respectively. The intrinsic T_2_ values and relative contributions of each peak are marked on each plot. For example, in Figure 1A at 288 K, the intrinsic T_2_ values for peaks 1 and 2 are 103 and 251 ms, respectively, and the relative contributions are 57 and 43% for the same peaks. In both figures, the T_2_ distributions exhibit two distinct peaks at different T_2_ values. As suggested before [14,15] for TGs, the two peaks may be the result of two distinct mobility populations of the protons on the chain, or inhomogeneous structural organizations with two different packing densities and intermolecular interaction intensities or types.

**Figure 2:**
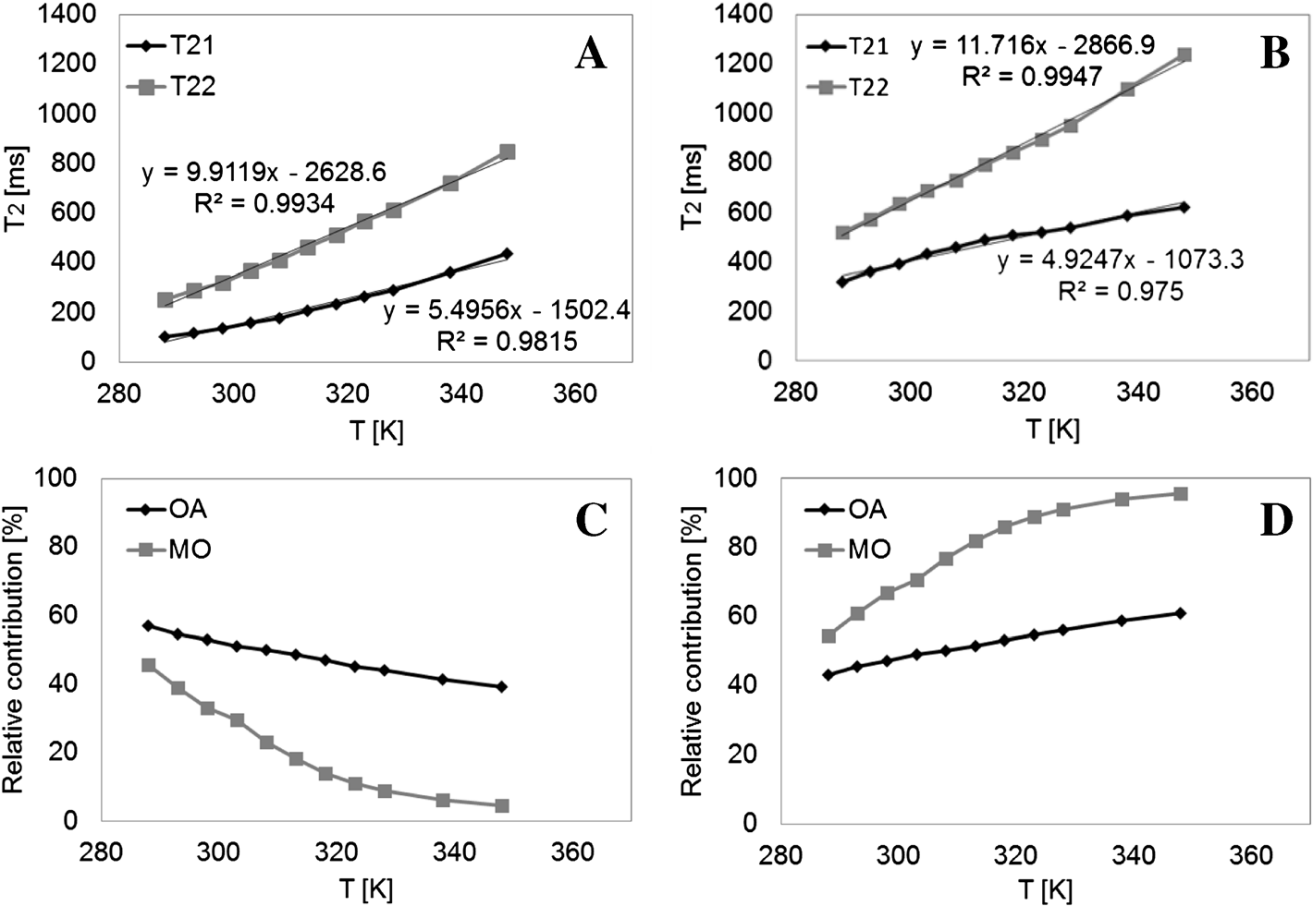
**Variation with temperature of intrinsic T**_**2**_ **values and relative contributions of OA and MO peaks.** Comparison of intrinsic T_2_ values of **(A)** OA and **(B)** MO; and relative contributions of **(C)** short (T_21_) and **(D)** long (T_22_) peaks of OA and MO.

Furthermore, pronounced alterations in the T_2_ distributions are found. When comparing two distributions for the same temperature, a shift in the T_2_ of the peaks of MO is observed towards higher values. Another interesting result is related to the change in the relative contribution of the peaks in the distribution as the temperature changes, which is mostly pronounced for MO. A closer look into the effect of temperature on the intrinsic T_2_ values and relative areas of each peak can be seen in Figure 2A-D. A highly linear increase with temperature of intrinsic T_2_ values can be observed for all peaks (R^2^ ≥ 0.98, Figure 2A and B). This suggests an increase in the mobility of different protons along the chain, or a change in the molecular organization with a change in intermolecular interactions towards the higher mobility peak (or population). Interestingly, the higher mobility peak (T_22_) is more affected by temperature, as it exhibits larger slopes compared to the low mobility peak (T_21_).

These differences are induced by small changes in the chemical structure of the lipid materials. Since both molecules (OA and MO) consist of the same tail (same length and position of double bond), the differences in this case are attributed to a methyl ester versus a carboxylic head group, which is responsible for a major intermolecular interaction of the chain with its neighbor.

As described in the introduction, the molecular organization of OA in the liquid has been extensively studied by Iwahashi's team ([17] and references therein). The stability of the interdigitated structure of OA was mainly attributed to their arrangement in a head-to-head conformation, driven by strong intermolecular hydrogen bonding of the carboxyl groups. In the case of esters, the arrangement of the heads is not obvious and depends on the polarity of the head group. Malkin [29] stated that methyl esters in the solid state behave in a weaker degree like the acids, crystallizing as dimer molecules with the polar groups together. It has also been suggested that a head-to-tail arrangement, where the polar groups in all layers have the same direction, can only be formed in cases of extremely weak polar forces, as in ethyl stearate [30]. In a study to determine end-to-end distances of liquid alkanes, Brady *et al*. [31] substituted one or two ends of the molecules with bromine atoms. They found that with a single substitution the chains lined up end to end so that the Br atoms seek maximum contact with each other, and concluded that this was due to stronger interactions between two polar Br than those between Br and a hydrocarbon chain end. This can be considered as analogous to the polar interactions of two ester groups. In a study of the crystal structure of methyl stearate, the authors suggested that the molecules form double sheets like the acids, probably due to polar forces between oxygen and carbon atoms [32].

**Figure 3:**
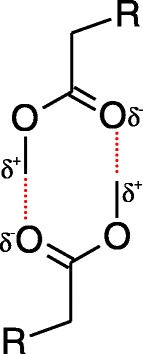
**Proposed configuration for the ester groups of two opposite MO molecules interacting with each other.** The resonance structures of the ester group result in weak interactions between the polarized hydrogen of the methyl carbon and the oxygen of the carbonyl on the opposite MO molecule. This molecular arrangement for MO would maximize the polar interactions.

We therefore assume the head-to-head conformation for the MO molecules in the liquid. Based on the crystal structure recently suggested for ethyl acetate [33], we would like to propose the configuration shown in Figure 3. Here the resonance structures of the ester group result in weak interactions between the polarized hydrogen of the methyl carbon and the oxygen of the carbonyl on the opposite MO molecule. This molecular arrangement for MO would maximize the polar interactions. The effect of this weaker interaction on their structure and mobility will be further discussed.

### X-ray measurements

In order to investigate whether short-range order exists for MO, and compare to the already-determined X-ray bands of OA, X-ray measurements were performed. X-ray patterns of liquids consist of one or several broad rings where the position of the maximum 2θ_max_ corresponds with high accuracy to the average intermolecular spacing, d. Iwahashi *et al*. [21] performed X-ray diffraction (XRD) measurements on several types of FAs, and found similar spectra for all the materials, consisting of mainly a large and sharp band around 0.14 nm^-1^ and a small and broad band around 0.03 nm^-1^. They suggested that the band around 0.14 nm^-1^ gives a measure of the spacing between adjacent molecules (short range), and the small band at around 0.03 nm^-1^ provides information regarding the long spacing of the plane made by the aligned molecules. In this study, XRD measurements on both materials showed only a single broad peak at around 0.14 nm^-1^, probably due to instrumentation differences. The 0.03 nm^-1^ peak was therefore measured using small angle X-ray scattering (SAXS) technology.

**Figure 4:**
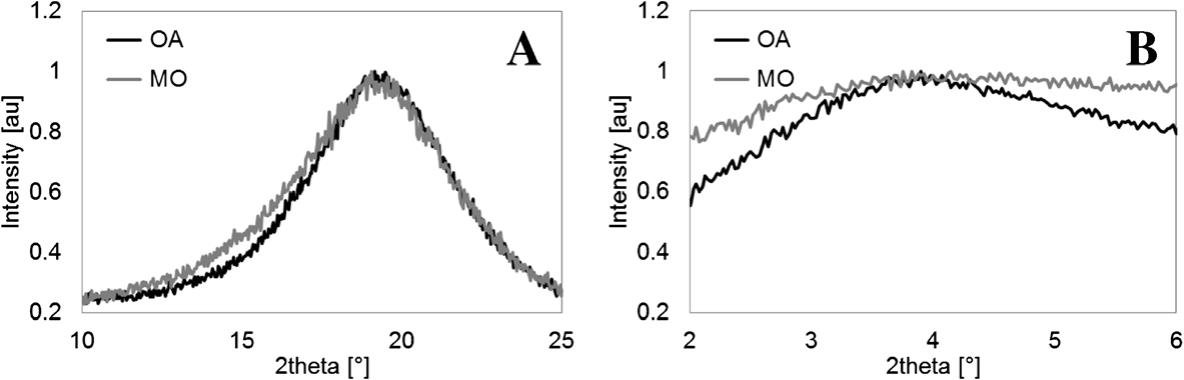
**X-ray spectra of OA and MO measured using (A) XRD and (B) SAXS at 298 K.** The peak at around 0.14 nm^-1^ (2θ ≈ 19.8°) is sharp for both materials, whereas the peak at around 0.03 nm^-1^ (2θ = 4.2°) is very broad and difficult to resolve, especially for the MO sample. SAXS: small angle X-ray scattering; XRD: X-ray diffraction.

Figure 4A and B show the X-ray spectra for OA and MO at 298 K using XRD and SAXS instruments, respectively. As shown, the peak around 0.14 nm^-1^ is sharp for both materials, whereas the peak around 0.03 nm^-1^ is very broad and difficult to resolve, especially for the MO sample. The broadness of the peaks suggests a lower degree of order, especially in the long-range spacing. The short- and long-range spacing values, d, derived from the corresponding spectra are summarized in Table 1.

**Table 1:** **Short- and long-range spacing, d, of OA and MO at 298 K**

	**d**_**OA**_ **[nm]**	**d**_**MO**_ **[nm]**
Short spacing (XRD)	0.459	0.460
Long spacing (SAXS)	2.383	2.531
		2.517^a^

^a^Long spacing measured at 263 K.

d_OA_ and d_MO_ are the short- and long-range spacings of OA and MO, respectively.

SAXS: small angle X-ray scattering; XRD: X-ray diffraction.

The short- and long-range spacings for the OA material were found to be in excellent agreement with those previously reported [21]. The short-range spacing of both materials was similar, probably due to similarities in the structure of the tails, leading to close interchain interactions. On the other hand, a larger long-range spacing was found for MO compared to OA, even when considering the distance from melting point (2.383 nm for OA at 298 K versus 2.517 nm for MO at 263 K). The difference in the long-range spacing originates from a larger distance between repeating planes. Considering the model proposed in this work for the head-to-head configuration of two opposite MO molecules (Figure 3), it would be fair to suggest that this is the result of the weak CH---O hydrogen bridges leading to larger distances between head groups. To be more specific, MO heads interact through weak polar interactions that form octagons (with a larger long-range spacing), while OA heads interact through hydrogen bonding, which makes hexagons (with a shorter long-range spacing).

The longer spacing between two MO molecules is a clear explanation for the reduced density and lower melting point of MO compared to OA (0.874 g/cm^3^ [34] and 253.1 K [35] versus 0.891 g/cm^3^ and 286 K (both taken from [19]) for MO and OA, respectively, with densities reported for 293 K). Both density and melting point parameters suggest a less efficient packing of MO.

**Figure 5:**
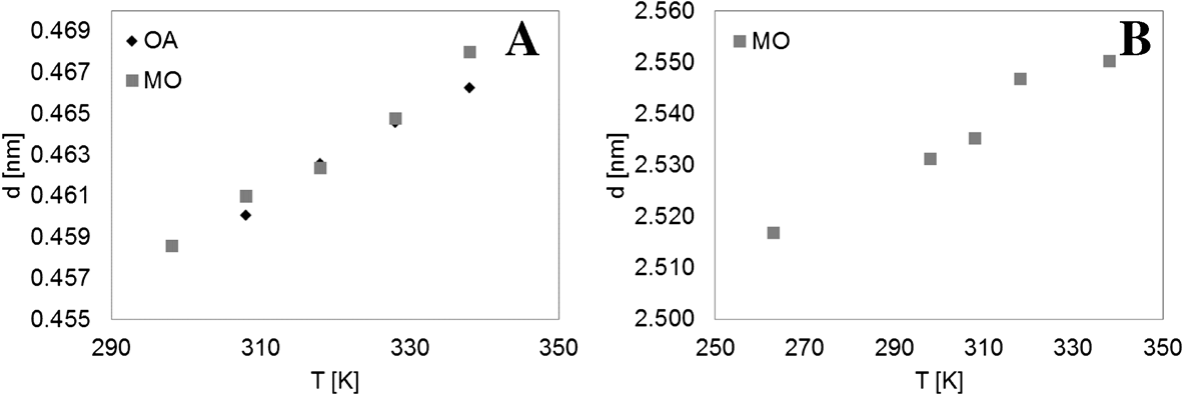
**Response of (A) short- and (B) long-range spacings, d, with temperature using X-ray measurements.** A similar increase in the short spacing with temperature for both OA and MO was observed. The long spacing for MO also increased with temperature and showed a moderate, close-to-linear increase from a temperature close to the melting point until 343 K.

To explore the effect of temperature on the short- and long-range spacings, further X-ray measurements were performed (Figure 5A and B). Iwahashi *et al*. [21] found that the long spacing in OA is constant regardless of temperature, whereas the short spacing increases with temperature. Our measurements show a similar increase in the short spacing with temperature for both OA and MO. The long spacing for MO also increased with temperature and showed a moderate, close-to-linear increase from a temperature close to the melting point until 343 K. These results imply a larger fluidity of MO compared to OA, because MO molecules separate both longitudinally and transversely from one another as the temperature is increased. Still, the appearance of both peaks for MO suggests that a degree of order, though on a smaller scale, is maintained even at high temperatures.

### Self-diffusion coefficients

**Figure 6:**
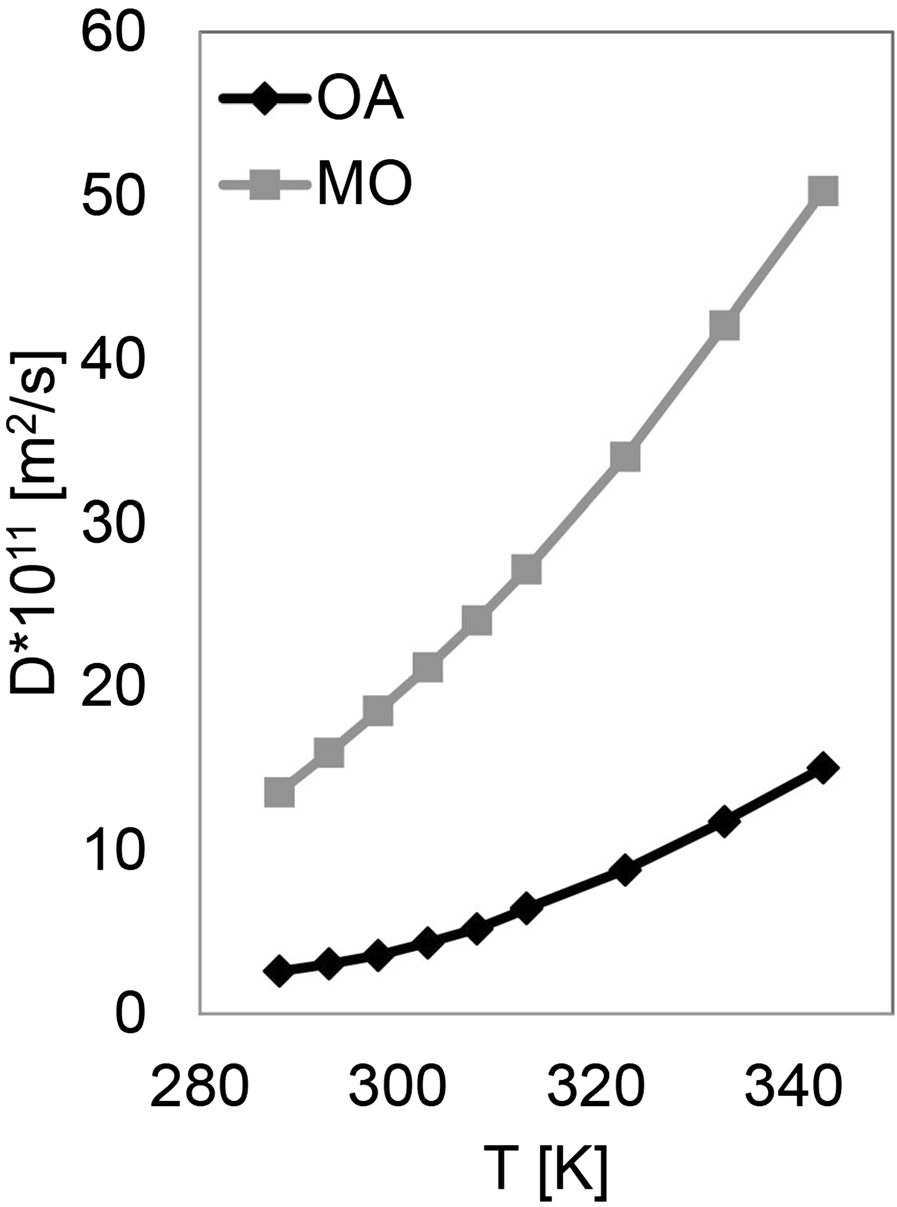
**Self-diffusion coefficient, D, of OA and MO at various temperatures.** MO exhibits larger D values compared to OA for all the temperatures, meaning that the translational movement of the ester is considerably larger than that of the acid.

The molecular structure of the molecules is expected to affect their translational movement. We therefore measured the self-diffusion coefficients, D, of OA and MO at various temperatures (Figure 6). As presented, MO has larger D values compared to OA for all the temperatures, meaning the translational movement of the ester is considerably larger than the acid. Both materials exhibit Arrhenius dependence of the form:

1 $$ D={D}_0 exp\left(-\Delta {E}_{app}/RT\right) $$

with apparent activation energies, ΔE_app_, of 27.0 and 19.5 KJ/mol for the OA and MO molecules, respectively. ΔE_app_ of OA is in fair agreement with the value reported by Yamamoto *et al*. [25] for the same molecule, and interestingly also with the value reported for liquid TGs [36]. These results suggest that OA molecular movement within its bulk liquid is considerably reduced compared with MO, and requires an equivalent amount of energy as a TG to initiate diffusion.

Dynamic viscosity measurements at different temperatures were performed on the MO sample (Table 2). These were then used to calculate the apparent hydrodynamic radius, r, from the Stokes-Einstein formula under a slip boundary condition (Equation (2) [17]):

**Table 2:** **Dynamic viscosity, η, of MO and apparent hydrodynamic radius, r, of MO and OA according to temperature**

**T [K]**	**η**_**MO**_ **[mPa s]**	**r**_**MO**_ **[nm]**	**r**_**OA**_^**a**^ **[nm]**
288	6.97	0.339	
293	6.03	0.332	0.315
298	5.27	0.336	
303	4.66	0.339	0.337
308	4.14	0.340	
313	3.71	0.341	0.330
318	3.34		
323	3.03	0.344	0.337
328	2.76		
333	2.52	0.345	0.345
338	2.32		
343	2.15	0.348	0.364
348	1.99		
353	1.85		
358	1.72		

^a^Calculated using the dynamic viscosities given in [19].

η_MO_ is the dynamic viscosity of MO; r_MO_ and r_OA_ are the apparent hydrodynamic radii of MO and OA, respectively.

2 $$ r=\frac{kT}{4\varPi \eta D} $$

where k is Boltzmann's constant, D is the self-diffusion coefficient, and η the dynamic viscosity. The apparent hydrodynamic radius of MO follows a very moderate linear (R^2^ = 0.80) increase with temperature (Table 2). This result agrees with the moderate increase with temperature of the short- and long-range spacings (Figure 5A and B). The r values of OA were also calculated using the dynamic viscosities given in [19].

Despite the large difference in ΔE_app_ of OA and MO, their apparent hydrodynamic radii were found to be very close (Table 2).

Iwahashi *et al*. [22] found that nonanoic acids in the liquid state remain as dimers even at 363 K. Based on this finding, they concluded that for normal FAs, dimers are the units in their intra- or intermolecular movements. Furthermore, they calculated the hydrodynamic radius of several normal FAs in the range of C8 to C18, and found that it decreases very slightly with increasing hydrocarbon chain. This suggested that the rotational (end-over-end) as well as transverse motion of each dimer is severely restricted, and that only a longitudinal translation (translational movement along molecular axes) is allowed.

Since the hydrodynamic radii r of OA and MO are very close, the large difference in D, and consequently ΔE_app_, can be attributed to their viscosity differences. It is well known that for long hydrocarbons, viscosity increases with number of carbons, due to a higher number of van der Waals interactions with adjacent molecules. Since both molecules have similar tails, one may rationalize that for the temperature range applied in this study, unlike the case of OA, which occurs as dimers, single MO molecules are the units in translational diffusion. Thus, for free motion, we would expect a larger difference in hydrodynamic radii r for single versus dimerized molecules (18 carbons versus 36 carbons). However, as stated before, the motion for long rod-like molecules is restricted to linear molecular movement; therefore, similar r values for the OA and MO molecules are to be expected.

### Segmental motion

**Figure 7:**
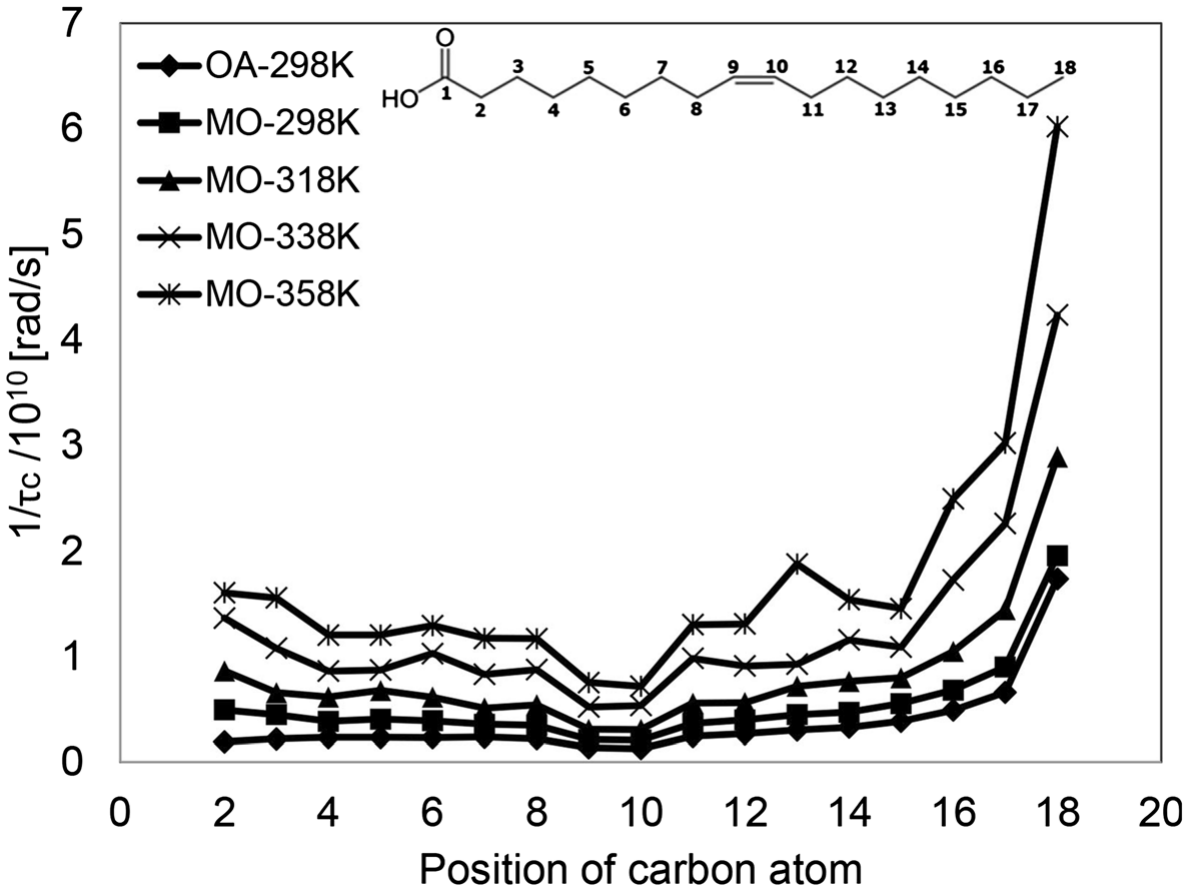
**Segmental motion of OA and MO at different temperatures.** Segmental motion through the reciprocal of the effective correlation time, 1/τ_c_, of each carbon can be calculated from T_1_, measured by ^13^C high field (HF)-NMR. OA was measured at 298 K, and MO at 298, 318, 338, and 358 K. The structure of OA along with designation of carbon numbers is shown for reference.

The spin lattice, T_1_, is likely to be correlated to the movement of the carbon atoms, i.e., segmental motion (specifically rotational tumbling and to a lesser extent translational and internal motion) in the molecule. The segmental movements at the end and near the end of the molecule are probably most important for the OA and MO molecules to find the spaces for their translational diffusion [21]. Of course, their close molecular arrangement and intermolecular interactions will work to hinder the segmental motion of some carbons. Segmental motion through the reciprocal of the effective correlation time, 1/τ_c_, of each carbon can be calculated from T_1_, measured by ^13^C HF-NMR. Figure 7 shows the 1/τ_c_ values of OA at 298 K and MO at 298, 318, 338, and 358 K. Assignment of ^13^C chemical shifts to the appropriate peaks was performed according to [37]. The results of 1/τ_c_ values of OA presented in this work are in excellent agreement with the results presented elsewhere [21].

A very close pattern can be seen when comparing the segmental motion of the different carbons on the OA and MO chains at 298 K. For both molecules, the rotational movement of the double bond carbons is considerably restricted due to stronger intermolecular interactions between the pi electrons of the double bond, and increases towards the end of the chains.

Nevertheless, two main differences are observed, which are both associated with the dissimilarities of head-to-head intermolecular interactions. The first is that all 1/τ_c_ values of the carbons along the chain of OA are smaller compared to the 1/τ_c_ of the carbons at the same position on MO. This is analogous to the shift of the entire distribution of MO to higher T_2_ values (Figure 1B), and can be attributed to the lower viscosity of MO. The second involves the rotational movements of the carbons closest to the head (C2 to C4). As suggested before, at a temperature of 298 K, OA molecules are almost entirely dimerized. This dimerization restricts the rotational movement, leading to pronounced rigidity compared to the methyl end. The head in MO, on the other hand, has a higher freedom of movement and is not tightly bonded, since 1/τ_c_ values decrease from the second carbon towards the double bond.

Nevertheless, there is a pronounced difference in 1/τ_c_ between the two ends of MO. Even though no strong hydrogen bonding exists in this ester, the polar interactions are sufficiently strong to limit the rotation of the head, so that it does not behave like the tail. This strengthens our initial assumption of polar interactions between the ester head groups.

As stated before, translational diffusion is probably initiated by the ends of the molecules. In the case of OA, dimers of two hydrogen-bonded molecules would move by the flipping of both tails on the dimer. MO molecules, on the other hand, would find available spaces for translational movement by very vigorous rotation of the tail, but also by wagging of the head.

1/τ_c_ values of all carbons in the MO chain increase with temperature, while maintaining the pattern described before. Interestingly, the 1/τ_c_ values of all the carbons on MO at 358 K (apart from C9 and C10) are higher than the 1/τ_c_ value of C17 at 298 K. This implies that the entire MO molecule moves around in the same vigorous manner as the tail does at 298 K. This is not the case for OA, judging by the values presented in [21]. Owing to the dimerization of the head, even at comparable high temperatures, the head and adjacent carbons do not reach the same degree of motion as the tail at 298 K.

### ^1^H and ^13^C HF-NMR chemical shift analyses

HF-NMR is a useful tool for identifying non-covalent interactions, as chemical shift is a sensitive measure of local chemical environment. Intermolecular interactions can be identified by changes in chemical shift. Therefore, to learn about the intermolecular interactions and their changes with temperature, we observed the change in chemical shifts of each proton and carbon on OA and MO, acquired using ^1^H and ^13^C HF-NMR, respectively.

**Figure 8:**
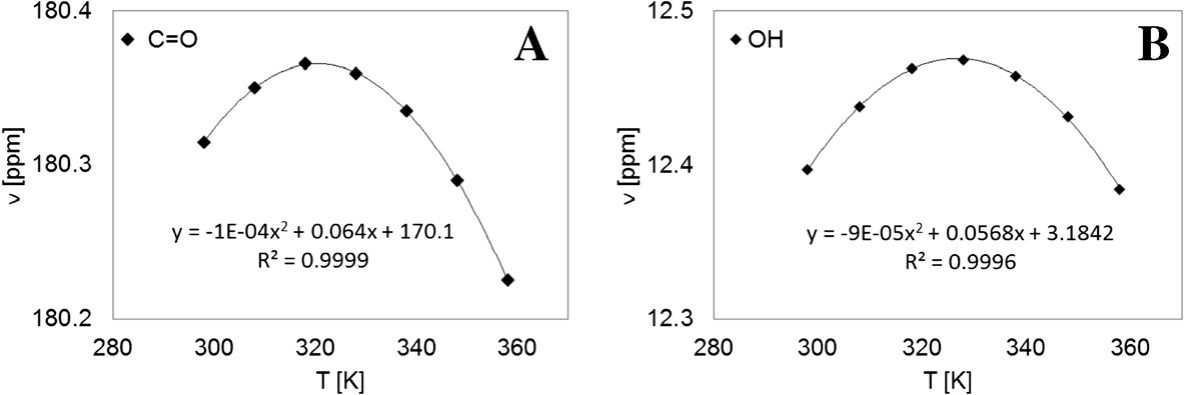
**HF-NMR chemical shift, ν, of the OA head (A)** ^**13**^ **C and (B)** ^**1**^ **H at different temperatures.** The carbon and proton on the carboxyl and hydroxyl groups, respectively, of OA, showed a varying behavior with increasing temperature, with maxima at approximately 320.5 and 327 K, respectively.

Unless otherwise stated, all chemical shifts moved downfield with increasing temperature. This can be explained by the increase of bond length with temperature, which requires less energy to cause nuclei inversion. In contrast, the carbon and proton on the carboxyl and hydroxyl groups, respectively, of the OA showed a varying behavior with increasing temperature, with maxima at approximately 320.5 and 327 K, respectively (Figure 8A and B for the carbon and proton, respectively). Iwahashi *et al*. [19] found that a discontinuous change takes place in some physical properties of the liquid OA around 328 K, and concluded that the liquid structure or molecular conformation changes into a more highly disordered one. This may serve as an explanation for the upfield increase of chemical shifts above this approximate temperature. Another possible explanation is the possibility that above a given temperature the H-bonding interactions between the carboxyl and hydroxyl groups is reduced by the mobility, so that the polar-electrostatic interaction in H-bonding is reduced, inducing an upfield shift. This is similar to the upfield shift observed on C1 of OA, as a function of pH [38], and on the OH group of octanoic acid as a function of temperature [26].

**Figure 9:**
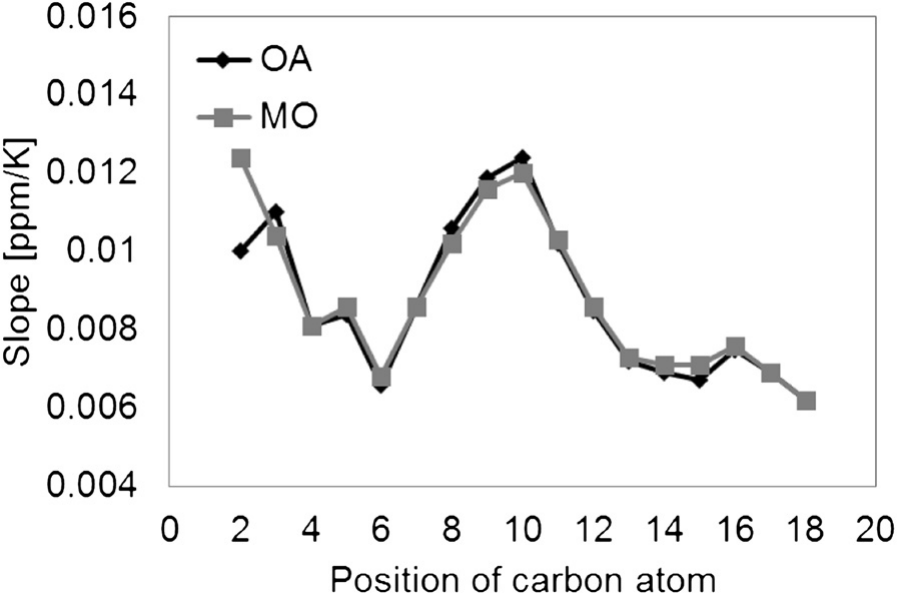
**The slopes for the** ^**13**^ **C HF-NMR chemical shift, ν, as a function of temperature for each carbon.** The slopes were calculated using linear regression of the change in ^13^C chemical shifts with temperature. Apart from C2, OA and MO have very similar slopes. On the other hand, the slopes differ to a great extent depending on their position.

The ^1^H and ^13^C chemical shifts for all other protons and carbons show a linear increase with temperature with different slopes. While the slopes for the different protons along the chain are very similar, those for the carbons differ to a great extent depending on their position (Figure 9). It is likely that the carbons with larger 1/τ_c_, meaning high rotational motion (Figure 7), are less affected by the changes in temperature and therefore exhibit small slopes. This is true for the carbons in the tail side of both OA and MO molecules. The double bond carbons C9 and C10, and the neighbor carbons, which show smaller 1/τ_c_ and low mobility, consequently show larger slopes. The C2 and C3 carbons on the head side of the molecules do not follow this behavior. For MO, although these carbons show a moderate increase in mobility with temperature (Figure 7), they show larger slopes than the carbons on the tail side. This apparent anomaly could be explained by the deshielding effect of the carboxyl group with temperature. The effect is induced through the C-C σ-bond, and is more effective in the α carbon than the β carbon, so that the slope of C2 > C3.

Conversely, in OA, the slope of C2 < C3. This can be explained by the varying behavior observed for the carboxyl group, which shows an upfield increase in chemical shift with temperature. Similar to MO, this shielding effect propagates trough the C-C σ-bonds, and is more intense in carbon α than β, explaining the C2 < C3 slope.

### ^1^H HF-NMR T_1_ and T_2_

**Figure 10:**
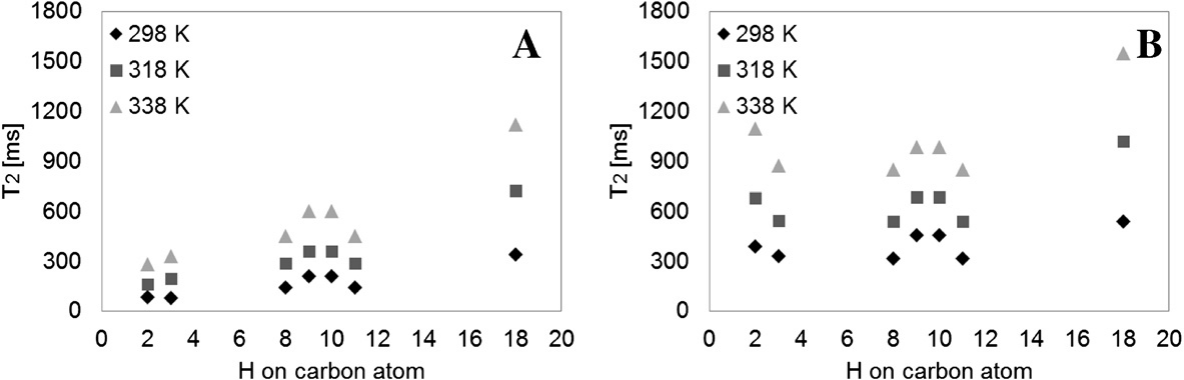
**Monoexponential T**_**2**_ **values of the resolved protons according to the position of the attached carbons.** Measurements of **(A)** OA and **(B)** MO were performed at 298, 318, and 228 K using ^1^H HF-NMR spectrometer.

In order to assign T_2_ values of different protons along the chains of OA and MO, the perfect echo sequence [39] was applied on a Bruker AVANCE III 600 MHz NMR spectrometer, at 298, 318, and 338 K. Peaks were assigned according to [40]. All protons exhibited a monoexponential behavior. Figure 10A and B show the monoexponential T_2_ values for the resolved protons according to the position of the attached carbon. The T_2_ values fit very well with the segmental motion presented in Figure 7, with the exception of the double bond protons. For all temperatures, the protons on MO have larger T_2_ values compared to the equivalent ones on OA. All the protons show a linear increase in T_2_ values with temperature. Interestingly, the T_2_ values of MO show a greater response with temperature, compared to OA. This is possibly comparable to the larger increase with temperature of the higher mobility peak (T_22_) and relative peak contributions of MO as seen in the T_2_ distributions (Figure 2A-D).

^1^H T_1_ values were also measured for OA and MO, on the 600 MHz NMR spectrometer, at 298 K. Table 3 shows the T_1_/T_2_ ratio of protons according to the position of the attached carbon for the two samples at 298 K. In the case of 2Πν_0_τ_c_ < 1, where ν_0_ is the Larmor frequency and τ_c_ is the correlation time, T_1_ ≈ T_2_ with T_2_ slightly smaller than T_1_. This is the case for non-viscous small molecules that exhibit fast rotation and tumbling. For larger molecules, rotation and tumbling are hindered, and an increase in T_1_ along with a decrease in T_2_ is expected. Based on this designation, MO exhibits liquid-like behavior, while OA shows a more hindered nature, with the highest rigidity presented for the head and double bond protons (Table 3).

**Table 3:** ^**1**^ **H T**_**1**_ **/T**_**2**_ **ratio of OA and MO measured using HF-NMR spectrometer at 298 K**

	^**1**^ **H T**_**1**_ **/T**_**2**_	
	**OA**	**MO**
C2	8.50	2.54
C3	7.59	2.65
C8/C11	4.48	2.23
C9/C10	5.87	2.71
C18	3.95	2.66

## Discussion

The only primary molecular difference between MO and OA is the methyl group on the acid moiety. This significantly affects the morphological structure of the material above its melting point. A key question at this point is whether MO in the liquid at the temperature range of this study has a molecular arrangement resembling that of OA. A molecular arrangement in this case would resemble to some extent that of a liquid crystal (as suggested for OA [19]), meaning molecules within this arrangement diffuse much like those in a liquid, while maintaining some degree of orientational and sometimes positional order as a function of the specific morphology. More specifically, this would mean that at least one molecular axis tends to point along a preferred direction as the molecules undergo diffusion [41].

Following this classification, it is suggested that both OA and MO possess a liquid crystal-like order, according to the apparent hydrodynamic radius, r, since for both molecules the rotational, as well as transverse, motion is to some extent restricted, and only a longitudinal translation is allowed. Still, the dimerization due to hydrogen bonding of the head groups in OA leads to a greater molecular rigidity compared to MO, and to a more efficient packing. Considering the head-to-head model interaction suggested in this paper, it is also reasonable to assume the quasi-smectic liquid crystal-like structure for MO, where heads are aligned next to tails in an interdigitated structure, similarly to the case of OA [19]. Evidence can be seen from the long-spacing (Table 1) of lateral planes, which is roughly equivalent to the length of a single MO molecule. In the case of adjacent heads the long-spacing is expected to be approximately double the one reported. However, the broader small angle peak acquired for MO (Figure 4B) suggests a less ordered structure in this respect.

It is also important to consider that the self-organization of these long rod-like molecules is temperature dependent, and may vary to a great extent as temperature increases further away from the melting point. Phase transition temperatures can give an estimation of intermolecular interactions and the degree of molecular order. Higher melting points are related to more efficient packing in the solid. A boiling temperature is required to break all intermolecular interactions present in the liquid. Crystallization is affected by the degree of order present in the liquid, such that more ordered molecules will crystallize at higher temperatures. The crystallization, melting, and boiling points of OA and MO according to the literature are shown in Table 4 (for OA the lower melting point for the α polymorph is referenced). The higher crystallization, melting, and boiling points of OA are an indication of the higher degree of intermolecular interactions and molecular packing arrangements compared to MO, both in the solid and in the liquid. Based on their chemical structure, this is clearly the effect of hydrogen bonding of the head groups in OA versus weaker polar interactions for MO.

**Table 4:** **Comparison of phase transition points of OA and MO**

	**Crystallization point [K]**	**Melting point [K]**	**Boiling point** ^**a**^ **[K]**
OA	277.7 [14]	286.0^b^ [14]	496.0 [42]
MO	232.5 [43]	253.1 [35]	474.0 [42]

^a^Measured at 1.333 kPa.

^b^The melting point for the α polymorph is referenced.

Interestingly, a significantly larger temperature hysteresis between crystallization and melting points exists for MO compared to OA (20.6 versus 8.3 K, respectively, calculated from Table 4). This suggests a smaller degree of order in MO, thus requiring a substantially reduced temperature to crystallize, since crystallization requires good molecular packing to reach an adequate entropy level.

Lastly, the task of assigning the molecular basis of the two peaks in the T_2_ distributions remains (Figure 1A and B). Based on the acquired information, we now refer to the original suggestions for the two peaks and offer more informed explanations.

### The two peaks are the result of inhomogeneous organizations with two different packing densities and intermolecular interaction intensities or types

Badmaev *et al*. [44] suggested a cluster model by which any liquid presents a micro-inhomogeneous medium, consisting of two dynamic components: ordered areas (clusters) and an inhomogeneous disorder matrix. Hernqvist [45] also proposed a dynamic model for liquid tristearin that consists of a lamellar liquid crystalline phase, where the size and orientation of the units vary with diffusion rates of the molecules and therefore with changes in temperature. This can be described as a transiently structured liquid with centers of organized structures forming and dissolving continuously, thus forming an equilibrium structure.

Following the cluster model, a reasonable assignment for the T_2_ distributions of OA and MO would be that the first peak (T_21_) consists of the molecules in the liquid crystal clusters and the second peak (T_22_) would be the result of the more liquid-like molecules. The molecular structure described before would therefore be responsible for the organization within the liquid crystal clusters, whereas the other liquid molecules in the amorphous morphology volumes would diffuse randomly throughout the sample volume with the molecular axes in rapid rotary-like movement. This kind of microstructural organization can be the result of structural memory coming from the solid structure, and can very well explain the two peaks.

Following this model, T_21_ would stand for the average T_2_ value for all protons inside the liquid crystal cluster, and T_22_ the average T_2_ value for the free more mobile ones. Since a very small difference exists between the two groups, T_21_ and T_22_ have close values at each temperature, although due to a more ordered nature and closer packing, T_21_ is smaller. As temperature increases, molecules from the cluster break up and transfer to the disordered matrix, as can be seen for the change in the relative contributions of the peaks. This pattern is more observed for MO, in the range of temperatures tested in this study. As suggested before, due to dimerization of the heads in OA, it has a denser arrangement and requires higher activation energy to initiate diffusion. As a result, the exchange of molecules between the two groups is considerably slower than that of MO, and the peak loss of T_21_ in OA is significantly less than in MO as the temperature is increased.

**Figure 11:**
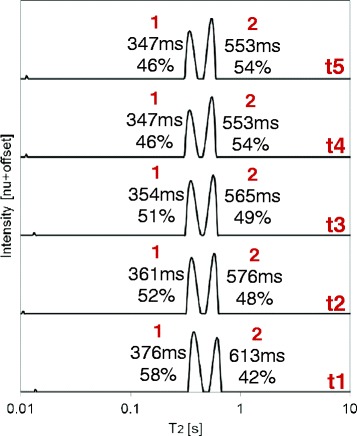
**Monitoring of the exchange of molecules between populations (possibly amorphous and liquid crystal environments) until stabilization.** Combined ^1^H low field (LF)-NMR T_2_ distributions of MO heated from 193 to 288 K, the temperature of measurement, at five increasing times (t1 to t5 according to the order of measurement). The first measurement (t1) was performed following 1 h of stabilization at 288 K. The relative contributions of each peak, in relation to other peaks and intrinsic T_2_ values, are shown on each plot. The relative contribution of the peaks changes in favor of the second peak as time progresses, until reaching a steady state.

A very interesting phenomenon that supports this model was observed in the T_2_ distributions, whose possible explanation may be monitoring of the exchange of molecules between amorphous and liquid crystal environments until stabilization. This can be seen for MO heated from 193 to 288 K, the temperature of measurement (Figure 11). Measurements were taken at five increasing times (t1 to t5 according to the order of measurement) until final stabilization was achieved at t4. As shown, the relative contribution of the peaks changes in favor of the second peak as time progresses, until reaching a steady state.

Pulse NMR has long been suggested as a tool for measuring solid fat content in partially crystallized fats. In the food industry, solid fat content values measured at different temperatures can be used to help predict important attributes such as mouth-feel and hardness. In this application the signals of both the solid (crystallized) fat and liquid oil are acquired. Possibly, with the current method, it is also conceivable to measure the ratio between the more rigid (clusters) and looser parts of the liquid. NMR and especially LF-NMR relaxometry would therefore be an excellent tool for monitoring changes in weak morphologies and/or interactions. This may be due to the low energy required to excite spin systems. The low frequency relaxation process in liquids concerns low energy processes, and can be explained by interaction of clusters.

### The two peaks are the result of two distinct mobility populations of the protons on the chain

Since temperature influences the self-organization of OA and MO, as suggested before, comparison of distributions would be made relative to the temperature of melting of each compound. Therefore, the comparison would be performed by subtracting the melting point of each compound (about 288 and 253 K for OA and MO, respectively) from the temperature of measurement. Following this rule, the MO distribution acquired at 288 K should be compared with the OA distribution acquired at 318 K, and so on. It can be seen (Figure 1A and B) that T_22_ is almost similar for the two materials (most pronounced for high temperatures), whereas T_21_ of OA is shorter than that of MO. This can indicate two populations: T_22_ is very close for both materials and can be assigned as the less restricted parts of the molecules; T_21_, on the other hand, is the more rigid part of the molecules, where OA is more restricted than MO (due to smaller values). Rigidity will lead to differences in intermolecular interactions, such that the more rigid parts have a close neighbor to interact with, leading to lower T_2_. The relative contribution of the peaks changes with temperature towards the less rigid peak. This is more pronounced for MO, since its head is freer to move compared to OA, as shown by the segmental motion and T_2_ values measured on a 600 MHz ^1^H HF-NMR spectrometer (Figures 7 and 10A, B, respectively). Peak assignments would therefore be as follows:

For OA, at intermediate temperature, the hydrogens close to the head, from C2 to C10 (18 hydrogens) are less mobile (T_21_ group) than the hydrogens from OH and from the tail, C11 to C18 (18 hydrogens, T_22_ group). The 1:1 ratio between these two groups is observed at 308 K. At higher temperatures, part of the head (C2 to C10 hydrogens) develops a mobility similar to that of the tail. At lower temperatures part of the tail hydrogens are in the head signal.

The assignment for MO is similar. At low temperatures, the C11 to C18 and OCH_3_ (20 hydrogens) are grouped in T_22_ and C2 to C10 (16 hydrogens) in T_21_. This is the ratio at 288 K. At higher temperatures, the T_2_ of part of the hydrogens from the head group "jumps" from the short to long T_2_. At 338 K, apparently, only the two olefinic hydrogens are part of T_21_ (6/94%).

**Figure 12:**
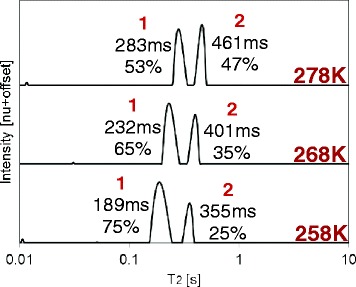
**Combined** ^**1**^ **H low field (LF)-NMR T**_**2**_ **distributions of MO at low temperatures (just above melting point).** This figure is an extension of Figure 1B. The relative contributions of each peak, in relation to other peaks and intrinsic T_2_ values, are shown on each plot. The change in T_2_ values of the peaks and distribution between populations is constant from the melting point and above.

In order to rule out the possibility that the large change in relative contributions of the peaks of MO, in response to an increase of temperature, is due to a loss of structural organization at temperatures far above the melting point, additional measurements were carried out at 258, 268, and 278 K (Figure 12, this is an extension of Figure 1B). Two peaks were once again observed for all temperatures, with similar trends of increasing T_2_ values and relative contributions with increasing temperatures, meaning the change in T_2_ values of the peaks and distribution between populations is constant from the melting point and above. This strengthens our assumption that the different response to temperature of OA and MO is due to differences in their chemical composition (head interactions). Following the comparison rule proposed before, the T_2_ distributions near melting should be compared (288 and 258 K for OA and MO molecules, respectively). Based on the peak assignment suggested before, it appears that at 258 K 75% of MO is rigid, though still more mobile than OA (both T_21_ and T_22_ have larger values than for OA).

Both theories for the assignment of peaks in the T_2_ distributions can be sustained by logical reasoning. However, the supporting data presented, especially ^13^C and ^1^H HF-NMR measurements, suggest that the two peaks are the result of two distinct mobility populations of the protons on the chain. An additional possible experiment to get a more conclusive choice between the models could be the use of partially deuterated OA and MO, for example, deuterating the carbons in the tail end (C11 to C18). If the T_2_ distribution remains unchanged, it would support the first model. However, a large reduction in the relative contribution and T_2_ value of the second peak would support the second model. Additional possible experiments would also involve measurement of several other standard FA and FAME materials of various chain lengths and degrees of unsaturation. Our research group has already initiated a thorough research design comprising these experiments. However, these are beyond the scheme of the current work and will be discussed in a separate publication.

The goal of this work was to explore the potential of ^1^H LF-NMR relaxometry for characterizing the molecular organization of lipids in the liquid phase. Although the T_2_ distributions acquired may well explain the cluster model of a microstructure arrangement and exchange of molecules between domains, the supporting measurements performed suggest otherwise. Still, bearing in mind that the mobility of the molecules is the direct outcome of their morphological structure, the differences in the molecular arrangement of OA and MO can be proposed by monitoring the differences in T_2_ distributions and peak area in response to a gradient of temperatures. This can be observed from the similarities in T_2_ distributions in relation to melting point. In this way, the large change in relative contribution of the peaks for MO suggests a less dense packing compared to OA. Ultimately, the dimerization of the head in OA compared to weaker polar interactions of the head can be clearly deduced from the increased intrinsic T_2_ values of MO.

Compared to other spectroscopic methods used in this study, ^1^H LF-NMR was found to be highly susceptible to low energy, weak intermolecular interactions, and aggregation of molecules. This technology is therefore suggested as a potentially important tool for the field of biodiesel. Several disadvantages have been suggested for the use of biodiesel in unmodified diesel engines, including cold weather operability and flow properties. In addition, a major problem exists with oxidative stability and the stability of biodiesel-petrodiesel blends, especially during prolonged storage. These physico-chemical properties are the direct outcome of the mobility and molecular structure of the fuel in the liquid. A possible application could be to test different additives to reduce dipolar interaction between MO heads. A small amount of additive could increase mobility and reduce viscosity, which would be easily observed by ^1^H LF NMR. The same could be applied to test the effect of biodiesels prepared from different feedstocks, meaning different FA profiles, and/or biodiesel-petrodiesel blends, and their differences in mobilities and packing. By reducing the temperature, going from the liquid down to the crystallization point, this technology may also be applied to test the initiation of crystallization and to monitor its progress. ^1^H LF-NMR can also be used for monitoring oxidation of biodiesels. We found an indication for this application in the course of our experiments on a sample of methyl linolenate. An additional peak at lower than usual T_2_ values was observed following prolonged storage.

## Conclusions

We have shown that OA and MO have both similarities and differences as seen in the ^1^H LF-NMR relaxation times and relative contributions according to temperature. These were attributed to a degree of liquid crystal-like order of both molecules that is temperature dependent, and that affects their ability to interact with close neighbors. Two suggestions have been made for the peaks: two distinct mobility populations of the protons on the chain; or a microstructural organization of the liquid with ordered areas and inhomogeneous disorder matrix. These properties allow for the material characterization based on parameters that contribute to important material properties in applications such as biodiesel fuels. This new application is of high potential to the field of biodiesel, and to other research and applied disciplines wherein relative weak interaction forces play an important part in physico-chemical characteristics.

## Materials and methods

### Materials

High purity (≥99%) OA and MO lipid standards were purchased from Sigma-Aldrich. The standards were used as received without further purification. Both standards were kept at 253 K between measurements, and heated from this temperature to the required temperature of measurement.

### ^1^H LF-NMR relaxometry and diffusiometry

All ^1^H LF-NMR measurements were performed on a 20-MHz minispec bench-top pulsed NMR analyzer (Bruker Analytik GmbH, Rheinstetten, Germany), equipped with a permanent magnet and a 10-mm temperature-controlled probe head. Measurements were first performed in the temperature range of 288 to 348 ± 0.03 K in 5 K steps. Prior to measurement, the samples were heated for minimum 1 hour and then allowed to equilibrate inside the instrument for 5 minutes. All measurements were performed on liquid standards (above melting point). For each temperature and sample the following parameters were optimized: receiver gain, magnetic field offset, detection angles, P90, P180, and homogeneity.

For MO, comparison of the data acquired at several temperatures starting from 193 or 253 K was performed, and similar distributions were found for the same temperature. However, the time to achieve stable results from 193 K was very long (several hours, especially for the higher temperatures). Therefore, MO was kept at 253 K between measurements, and heated from this temperature to the required temperature of measurement.

Determination of spin-spin relaxation (T_2_) was performed using a CPMG (Carr, Purcell, Meiboom, and Gill [46,47]) pulse sequence. Values of τ of 0.4 and 1.125 s and recycle delays of 4 and 6 s were used for the OA and MO samples, respectively. Additional CPMG measurements of MO were performed at close to melting temperatures (258 to 278 ± 0.03 K in 10 K steps). Acquisition at low temperatures for MO was performed with a τ of 0.4 to 0.5 s and a recycle delay of 5 s. For all the analyses, 32 scans were accumulated and 8,000 echoes were acquired. Data was acquired in magnitude mode due to better repeatability and stability of results, and further analyzed using the primal-dual interior method for convex objectives (PDCO) optimization algorithm with α_2_ = 0.5, as described in [7]. CONTIN (software application for inverse Laplace transformation of LF-NMR relaxometry data available in minispec) was also used for analyzing the acquired CPMG data, in order to compare distributions with PDCO. The mathematical formulation of CONTIN is described in [48]. Both methods exhibited very similar T_2_ distributions, although better repeatability and stability was found with PDCO analyses.

The self-diffusion coefficient, D, was determined by the pulsed-field gradient spin echo (PFGSE) method [49]. The PFGSE sequence was used with 16 scans, a τ of 7.5 ms, and a recycle delay of 6 s. Typical gradient parameters were Δ of 7.5 ms, δ of 0.5 ms, time between the 90° pulse to the first gradient pulse of 1 ms, and g of 1.6 T/m. A water sample (1.25 g/L CuSO_4_) was used for calibration. The D values of water were taken from [50]. Each reported value is the average of a minimum of ten measurements.

### High field ^1^H and ^13^C-NMR chemical shift, spin-spin, and spin-lattice relaxation time

The high field ^1^H and ^13^C-NMR measurements were performed on a BRUKER AVANCE III operating at 600 MHz for ^1^H nuclei and 150 MHz for ^13^C. Prior to measurement, samples were heated for a minimum of 10 minutes and added in a 5-mm NMR tube. For the lock signal, a closed 1-mm capillary tube, filled with D_2_O, was added to the sample. The non-spinning samples were allowed to equilibrate inside the instrument for 15 minutes after reaching the set temperature. Before each measurement, shimming was optimized using automated and manual procedures. The chemical shifts, in parts per millions (ppm), were obtained without a reference signal. The ^1^H and ^13^C NMR spectra were obtained using 4 and 8 scans and recycle delays of 30 s and 120 s, respectively.

The ^1^H and ^13^C longitudinal relaxation times, T_1_, were measured using the inversion recovery method [51]. The ^1^H transverse relaxation time, T_2_, was measured using a modified (perfect echo) CPMG pulse sequence (PROJECT - Periodic Refocusing of J Evolution by Coherence Transfer) that resulted in spectra without J modulation [39]. The calculations of T_1_ and T_2_ were carried out with the subroutines included in the TOPSPIN 3.2 software package.

^13^C HF-NMR spin-lattice relaxation of a protonated carbon is overwhelmingly dominated by dipole-dipole interactions with the attached protons [21]. T_1_ is therefore related to the number of directly bonded hydrogens, N, and the effective correlation time, τ_c_, for the rotational movement of the carbon atoms in the object molecule. Thus, T_1_ is approximately given in terms of N and 1/τ_c_:

3 $$ {T}_1=\frac{r_{CH}^6}{N{\hslash}^2{\gamma}_C^2{\gamma}_H^2}\left(\frac{1}{\tau_C}\right) $$

where ħ is Planck’s constant and γ_C_ and γ_H_ are the gyromagnetic ratios of ^13^C and ^1^H, respectively. Here, r_CH_ is the C-H distance, usually about 0.109 nm, and the reciprocal of the effective correlation time, 1/τ_c_, represents the magnitude of the segmental rotation for the carbon atom at a different position.

### X-ray methods

In this study, XRD and SAXS techniques were used for measuring the short- and long-range spacing between adjacent molecules, respectively.

XRD data was collected on a Panalytical Empyrean Powder Diffractometer equipped with a position-sensitive (PSD) X’Celerator detector using Cu K_α_ radiation (λ = 0.154 nm) and operated at 40 kV and 30 mA. The usual Bragg-Brentano θ/2θ geometry was employed. θ/2θ scans were run for 15 minutes in a 2θ range of 2 to 35° with step equal to about 0.0167°. Measurements were performed at a range of 298 to 338 K in 10 K steps.

SAXS measurements were performed on a SAXSLAB GANESHA 300-XL (Skovlunde, Denmark) instrument. Cu K_α_ (λ = 0.154 nm) radiation was generated by a Genix 3D Cu source (operated at 47 mV and 0.55 mA) with integrated monochromator, 3-pinhole collimation, and a two-dimensional Pilatus 300 K detector. The distance between the sample and detector was 350 mm. The q range was between 0.0012 to 0.067 nm^-1^. OA was measured at 298 K, for comparison with the literature, and MO was measured at several temperatures, including 263 K, 298 to 318 K in 10 K steps, and 338 K.

### Dynamic viscosity

Dynamic viscosity measurements of MO were performed on an AR 2000 Rheometer (TA Instruments), on a double-gap Peltier cylinder system in steady-state flow mode, in the temperature range of 288 to 358 K. For each temperature, 10 points were acquired in the range shear rate 10 to 300 s^-1^, and the average was reported.

## Abbreviations

CPMG: Carr, Purcell, Meiboom, and Gill
FA: fatty acid
FAME: fatty acid methyl ester
HF-NMR: high field nuclear magnetic resonance
LF-NMR: low field nuclear magnetic resonance
MO: methyl oleate
OA: oleic acid
PFGSE: pulsed-field gradient spin echo
SAXS: small angle X-ray scattering
TG: triglyceride
XRD: X-ray diffraction
